# Mechanically Robust, Inkjet-Printable Polymer Nanocomposites with Hybrid Gold Nanoparticles and Metal-like Conductivity

**DOI:** 10.1021/acsami.4c04692

**Published:** 2024-06-11

**Authors:** Michael A. H. Klos, Lola González-García, Tobias Kraus

**Affiliations:** † INM—Leibniz Institute for New Materials, Campus D2 2, 66123 Saarbrücken, Germany; ‡ Saarland University, Colloid and Interface Chemistry, Campus D2 2, 66123 Saarbrücken, Germany; § Saarland University, Department of Materials Science and Engineering, Campus D2 2, 66123 Saarbrücken, Germany

**Keywords:** nanocomposite, printed electronics, sinter-free, adhesion, conductive

## Abstract

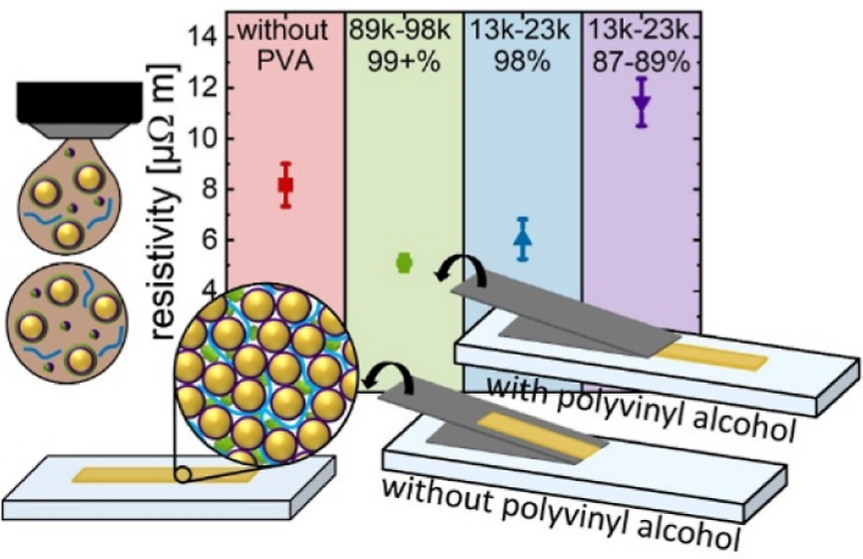

Hybrid core–shell nanoparticles with metal cores and conductive polymer shells yield materials that are sinter-free and highly conductive but mechanically weak. Conventional composites of such nanoparticles are electrically insulating. Here, we introduce microscale phase-separated nanocomposites of hybrid gold-PEDOT:PPS particles in insulating poly(vinyl alcohol) (PVA). They combine electrical conductivities of up to 2.1 × 10^5^ S/m at 10 vol % PVA with increased mechanical adhesion on polyethylene terephthalate and glass substrates. We studied the effects of the PVA molecular weight, hydrolyzation degree, and volume fraction. Composites with 10 vol % highly hydrolyzed PVA at a *M*_W_ of 89–98 kDa had the highest conductivities and stabilities; highly hydrolyzed PVA even increased the conductivity of the hybrid particle layers. We propose the formation of hydrogen bonds between PVA and PEDOT:PSS that lead to demixing and the formation of stable, structured composites. Finally, we demonstrated the inkjet-printability of inks containing PVA in water with viscosities of 1.6–2.0 Pa s at 50.1 s^–1^ and prepared bending-resistant electrical leads.

## Introduction

Printed conductive metal structures are suitable for sensors,^1,2^ RFID antennas,^3^ and other devices. Printing enables material-efficient application and is possible via dispensing,^4^ inkjet printing,^5^ aerosol jet printing,^6^ and other techniques. High-resolution printing of conductive structures requires inks with particulate components^7^ that are sintered after printing^8^ or with molecular precursors, such as metal complexes or metal ions,^9^ that are decomposed after printing. Their applicability is limited if sintering temperatures exceed the substrate stability or deform the structures.

An interesting alternative to thermal sintering are hybrid particles, such as CdSe@ZnS quantum dots with an organic block copolymer shell that Zorn et al. and Kwak et al. used for light-emitting devices.^10,11^ Gold and silver particles have been used as cores of hybrid conductive nanoparticles (hCNPs) covered by a semiconductor polymer.^12−14^ Escudero et al. coated gold nanoparticles with the conductive poly(3,4-ethylenedioxythiophene) polystyrenesulfonate (PEDOT:PSS) via a ligand exchange from cetrimonium bromide (CTAB)-stabilized gold nanoparticles. The conductive ligands bridged the gap between the metal cores and made the films conductive immediately after solvent evaporation.

The mechanical stability of metal films depends on their thickness, grain size, and adhesion.^15,16^ Defect-rich or porous metal films tend to be weaker in strength.^17^ The hybrid metal–polymer films discussed above are mechanically weak and require additional protection, which complicates the device design. On the other hand, nanocomposites that combine a nanoscale inorganic component with a polymer can have mechanical properties that exceed those of the polymer.^18,19^ The high surface-to-volume ratio of certain nanoscale fillers endows mechanically tough and scratch-resistance films with good adhesion.^20−22^ For example, established nanocomposites with silica nanoparticles at volume fractions of 40–60 wt % in polysiloxanes are suitable as scratch-resistant coatings on PMMA.^23^ Conductive nanocomposites have been created by hot pressing of conductive silver nanoparticles (1.3–15.6 vol %) in polyetheretherketone with an electrical conductivity of 6.7 × 10^4^ S m^–1^ at the percolation threshold of 10.8 vol % silver nanoparticles.^24^

Here, we show that the nanocomposite concept can be extended to printable, electrically conductive materials with a high fraction of electrically hCNPs. We used low polymer contents of 5–20 vol % poly(vinyl alcohol) (PVA) that permeate the volume and interact with the hCNPs. The result was a post-treatment-free nanocomposite that combines the good conductivity of hCNP with improved mechanical stability.

This work is structured as follows: first, we report on the colloidal stability and shelf life of hCNP–PVA dispersions. We then report the effect of the PVA fraction on the conductivity of drop-casted hCNP nanocomposites with a possible phase separation. We then study the mechanical properties of nanocomposites with PVA fractions below 20 vol % as a function of molecular weight and degree of hydrolyzation. We formulate inkjet-printable low-viscosity inks, print thin-film electrodes with a PVA fraction of 10 vol %, and quantify their conductivity, mechanical adhesion, bending resistance, and stretchability.

## Results and Discussion

Nanocomposite films of hCNP and PVA were prepared from liquid inks by drop-casting and inkjet printing. The PVA matrix is meant to enhance the mechanical properties by forming hydrogen bonds between hCNPs, PVA, and the substrate, but it is insulating and can reduce the conductivity through tunnel barriers between the individual hybrid particles. The desirable interaction of PVA and the hCNPs can lead to unwanted destabilization of the ink dispersion, too.

First, we investigated the influence of PVA on the colloidal stability of dispersed hCNPs. We used UV–vis spectroscopy to detect PVA adsorption and PVA-induced agglomeration. Nanocomposite films were then prepared at PVA volume fractions below 30 vol % to ensure percolation, a fundamental requirement of conductivity. The effects of polymer fraction, polymer molecular weight, and degree of hydrolyzation on the mechanical and electrical properties of the nanocomposites were quantified using drop-casted films. Films that combined good conductivity with mechanical robustness were then selected, and we created inkjet inks with corresponding compositions. Test structures were printed on glass, PET, and polyurethane (PU) substrates and tested for electrical resistivity, adhesion, bendability, and stretchability.

### Colloidal Stability of Inks Containing PVA and hCNPs

Printing electronics with Drop-on-Demand technology requires colloidally stable inks to prevent agglomeration, nozzle clogging, and particle settling. Literature reports that PVA can adsorb onto AuNPs and stabilize them.^25,26^

Other researchers used PVA as a shape-directing agent in the synthesis of gold nanoparticles and found effects on particle size and the ratio between polymer and gold by forming hydrogen bonds between different PVA molecules.^25^ We investigated whether such interactions exist between PVA and our hCNPs and whether they negatively affect the colloidal stability.

The hCNPs used in this work carried a shell of covalently attached PEDOT:PSS on a gold core (47 nm diameter) and were dispersed in water. They were prepared following published routes by Escudero et al. 2021 and formed pure layers with a conductivity of 44.2 μΩ m some minutes after printing.^13^

**Figure 1:**
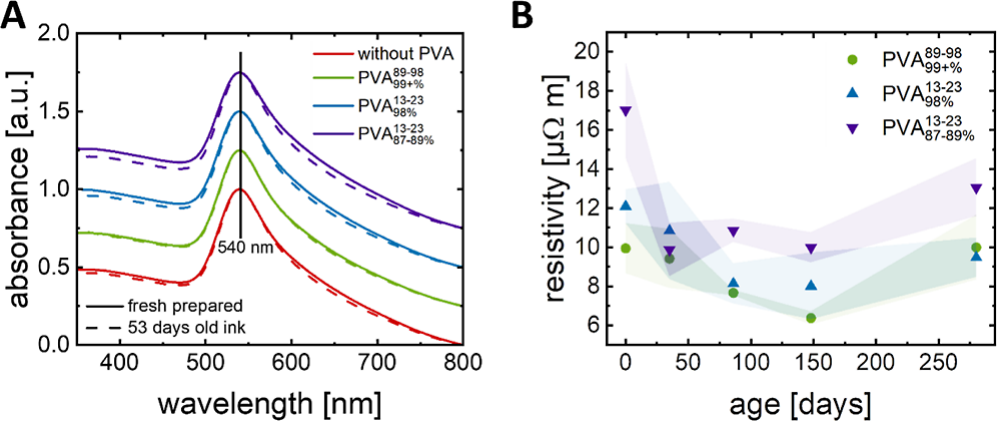
Stability of inks containing hybrid particles and poly(vinyl alcohol) (PVA) in water. (A) UV–vis spectra of freshly prepared (solid lines) and 53 day (interrupted lines) old inks. (B) Resistivities of drop-casted samples prepared from freshly prepared, 35, 86, 148, and 280 day old inks after 1 week of drying. Standard deviations of the mean from three to four samples are given as shaded colors. The PVA content in the dry film was 10 vol %. The molecular weight of PVA_99+%_^89–98^ was between 89,000 and 98,000 and 99+% hydrolyzed. PVA_98%_^13–23^ has a molecular weight between 13,000 and 23,000, 98% hydrolyzation, and PVA_87–89%_^13–23^ has a molecular weight of 13,000–23,000 and 87–89% hydrolyzation.

We investigated the colloidal stability of hCNPs in aqueous dispersions containing 0.726–0.742 wt % PVA per unit mass of gold (cf. Nanocomposite Preparation). The surface plasmon resonance (SPR) peak of the gold core at 540 nm (Figure 1A) is sensitive to particle size and dielectric environment.^27^ Spectra of dispersions recorded immediately after PVA addition and after 53 days of storage at room temperature did not show significant changes of SPR position or amplitude in any of the samples, confirming the absence of agglomeration in that period (Figure 1A). Agglomeration would cause a broad rise at wavelengths above our SPR wavelength.^28^

The unchanged SPR peak shape and position at 540 nm suggest an unchanged dielectric environment for the hCNPs. PVA did not replace PEDOT:PSS, apparently; such replacement would considerably reduce the electron density around the gold compared with the conjugated PEDOT:PSS and cause an SPR shift. We attribute this stability to the stronger affinity of the thiophene sulfur of PEDOT to gold than to the hydroxyl groups of PVA. It is conceivable that the sterically demanding PEDOT:PSS shields the gold particles, too, providing an additional kinetic barrier to replacement. It is also conceivable that the structure of the PEDOT:PSS shell changed and that a part of it was removed but that the overall thickness and dielectric properties of the shells remained.

In conclusion, we found that hCNPs dispersions remain colloidally stabilized after adding PVA. The following examines the long-term stability of PVA-containing inks.

### Long-Term Stability of PVA–hCNP Inks via Electrical Conductivity

We tested the shelf life of the PVA–hCNP inks with 10 vol % PVA (dry solid fraction) by storing them at room temperature for 35, 86, 148, and 280 days. Films were then prepared by drop-casting 2 μL of each ink on plasma-activated glass slides (cf. Film Deposition). Film thicknesses were determined by confocal microscopy and resistances by linear fits of *I*–*V* curves obtained in four-point-probe geometry. The calculated resistivities (Figure 1B) were in the range of 6.4–17.0 μΩ m for all used PVAs (PVA_99+%_^89–98^, PVA_98%_^13–23^, and PVA_87–89%_^13–23^), with variances that were likely dominated by the heterogeneous thicknesses of the drop-casted samples.

Figure 1B indicates the acceptable shelf life of the inks. The observed differences in resistivity are likely due to differences in the porosity or polymer distribution in the different materials. Differences in the interactions between different PVA types and the PEDOT:PSS shell probably lead to different microstructures of the layers. The unexpectedly high resistivity of the fresh sample containing PVA_87–89%_^13–23^ seems to be an artifact of sample preparation, while the systematically lower resistance of samples with PVA_99+%_^89–98^ suggests that a higher molecular weight or hydrolyzation degree systematically fosters microstructures with higher conductivity. The intermediate conductivities of PVA_98%_^13–23^ suggest a strong role of hydrolysis.

Note that the resistivities of all samples were in the range of 9.5–13.1 μΩ m after 280 days with a relatively weak degradation. We conclude that the shelf life of our PVA-containing hCNP dispersions was at least 53 days, sufficient for application.

### Electrical Conductivity of PVA–hCNP Nanocomposites as a Function of PVA Fraction

**Figure 2:**
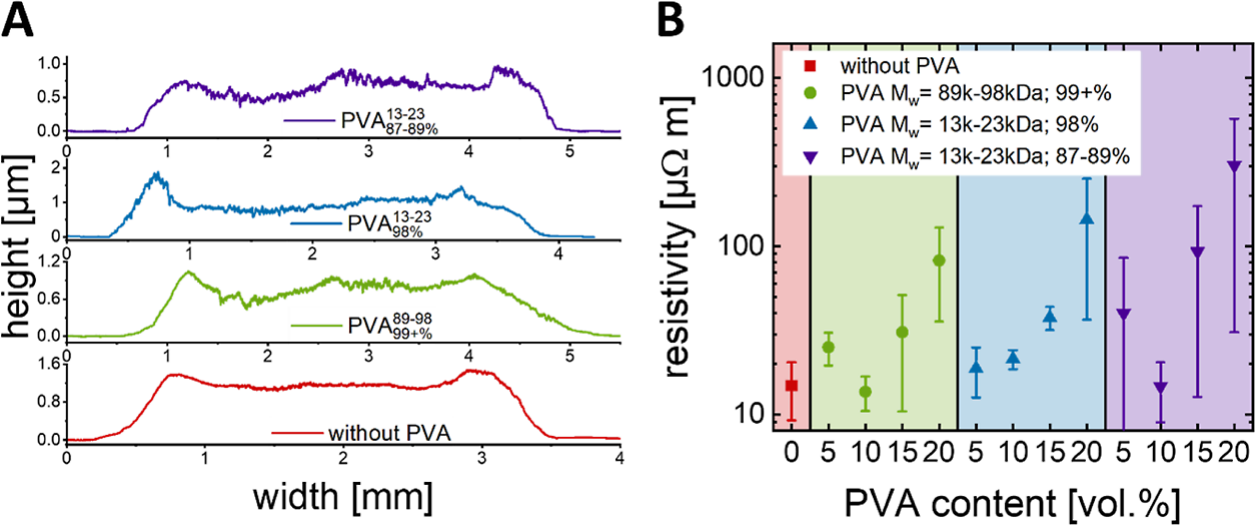
Profiles and resistivities of drop-casted nanocomposite films on PET with different polymer volume fractions of the solid and overall thicknesses in the ranges of 0.4 and 1.0 μm. (A) Typical height profile. (B) Average resistivities of three to four samples, with ±1 standard deviation indicated as the error bar.

We quantified the impact of PVA on the conductivity of the dried composites as a function of the solid polymer volume fraction and type of PVA. Inks with four concentrations of all PVA types were casted as lines on glass and PET foils. The samples were electrically characterized in a four-point-probe configuration, thicknesses were measured with confocal microscopy, and resistivities were calculated (cf. sections on Film Deposition, Characterization, and Long-Term Stability of PVA–hCNP Inks via Electrical Conductivity). We found thicknesses from 0.4 to 1.0 μm on PET for all samples, with average profiles calculated from individual measurements shown in Figure 2A. Average heights and widths were used to determine cross-sectional areas and calculate resistivities (Figure 2B). The resistivities were in the range of 14.8 ± 5.6 μΩ m (without PVA), 13.6 ± 3.2 μΩ m (10 vol % of PVA_99+%_^89–98^), 21.4 ± 2.8 μΩ m (10 vol % of PVA_98%_^13–23^), and 14.7 ± 5.7 μΩ m (10 vol % of PVA_87–89%_^13–23^). Resistivity increased when adding 5 vol % PVA but sometimes decreased when adding 10 vol % PVA (Figure 2B). PVA contents above 10 vol % always led to significant increases in resistance and a greater spread of resistances across different samples.

A part of the resistivity differences between samples stem from batch-to-batch variations in the inks. Separate experiments (results not shown here) indicated that the conductivities of films prepared with the same method from different batches varied in resistivities by a factor of 1.5–3.9, probably due to variations in the PEDOT:PSS dispersion and the ratio of PEDOT:PPS to gold in the hybrid inks. The addition of 10 vol % of PVA_99+%_^89–98^ consistently led to constant or even increased conductivities.

We found a clear effect of the PVA type on resistivity. All PVA types increased the resistivities by factors between 5 and 20 when added at 20 vol %. PVA_87–89%_^13–23^ always caused the largest and PVA_98%_^13–23^ the second-largest changes. The addition of PVA_99+%_^89–98^ at 10 vol % even reduced the resistance.

We performed additional resistance tests on the effect of water. Drop-casted samples (10 vol % PVA) were immersed in Milli-Q water for 1 h. They were taken from the vial, excess water was removed, and the resistances of the swollen layer were tested. All samples remained conductive (increase in resistance by a maximum of 150%) but became mechanically softer.

Increased resistances in percolating composites are expected when increasing the fraction of an insulating phase. The relations have been studied theoretically by Wang et al. considering both tunneling and ohmic transport in composites of randomly distributed gold nanoparticles in a polymer matrix as a function of particle size and particle content.^29^ Their model predicts lower conductivities for composites with fewer particles and higher polymer content.

In summary, conductivity measurements indicate that PVA contents of below 15 vol % do not necessarily increase resistivity. It is possible to use PVA_99+%_^89–98^ at 10 vol % as a mechanically reinforcing matrix without degrading conductivity and at up to 15 vol % if a reduction of 52% in conductivity is acceptable.

### Phase Separation Due to Hydrogen Bonds between PVA and PSS

The decreased resistivity at low PVA fractions that we find indicates a deviation from random mixing between the insulating and conductive phases. The strong effect of hydrolyzation and the weak effect of molecular weight suggest demixing that depends on the degree of interaction of PVA with PEDOT:PSS as an underlying mechanism.

Interactions of hydroxide-containing polymers with PEDOT:PSS have been described in the literature. For example, Chen et al. used microscale crack analyses during tensile stretching to study the hydrogen bond network in cellulose/PVA/PSS films and found that the network improved mechanical properties.^30^ Wang and co-workers prepared PVA–PEDOT:PSS composite fibers and quantified hydrogen binding between PVA and PSS via a shift of the hydroxyl stretching band to smaller wavenumbers of the FT-IR transmittance peak.^31^ They explain the miscibility of the two components and an improvement in fiber properties with the interactions of PVA and the sulfonate group of PSS.

The addition of PVA likely leads to the formation of separated phases of conductive PEDOT-stabilized gold nanoparticles and a nonconductive PVA–PSS combination. This increases the conductivity by decreasing contact resistances between hCNPs in one phase. In a related case, Fallahzadeh and co-workers reported that the conductivity of pure PEDOT:PSS films was increased by treatment with alcohol and suggested conformational changes and phase separation as mechanisms.^32^ Zhang et al. reported that on PEDOT:PSS-coated particle films, PSS segregated and formed a top layer.^33^

**Figure 3:**
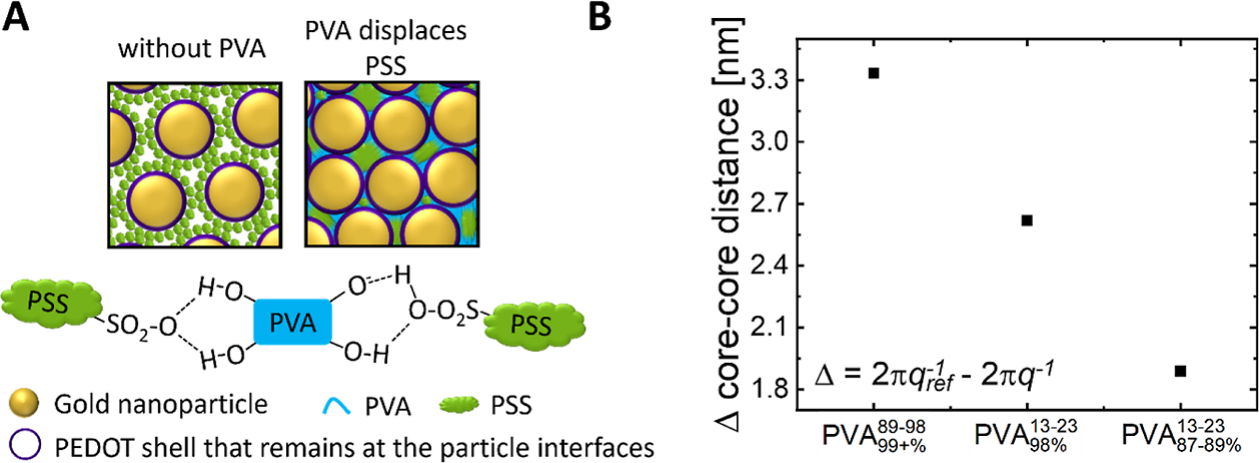
Microscale phase separation upon the addition of PVA to the hybrid nanoparticle films. (A) Illustration of the reconstructed hCNP–PVA microphases and the proposed interactions between PVA and PSS that drive segregation and reduce the distances between the conductive components. (B) Small-angle X-ray scattering (SAXS) results indicate that the reduction of core–core distances depends on the molecular weight of the PVA and its degree of hydrolyzation, which is consistent with the proposed phase separation model.

We suggest a phase separation between PVA–PSS and PEDOT, as illustrated in Figure 3. The weakly attached PSS interacts with PVA and leaves the hCNPs, forming a separate phase. This explains the different trends that we experimentally observe: The addition of PVA at larger volume fractions (>10 vol %) increases resistivity because it disrupts conductive networks. The addition of low-volume fractions (5 vol % and below) disrupts some parts of the networks but does not yet induce phase separation (depending on PVA type). The addition of 10 vol % of certain PVA types induces the phase segregation of PVA–PSS, removes insulating PSS from in between hCNPs, and reduces the resistivity of the particle contacts. A network of PEDOT-coated gold nanoparticles forms during drying that is mechanically stabilized by a PVA network. We propose that PSS forms a distributed phase that does not impede electrical conductivity, as illustrated in Figure 3A.

The different resistivities as a function of PVA type are a consequence: a larger density of hydrogen bonds for highly hydrolyzed PVA increases the number of interactions through hydrogen bonds. Longer PVA chains or lower degrees of hydrolysis thus aid in demixing in water.^34,35^ This model also explains why the resistivities of samples with lower degrees of hydrolysis had larger variability: such PVA chains form fewer hydrogen bonds per volume, which reduces the driving force for demixing of PVA–PSS phases and results in smaller volume fractions of the microscopically segregated material. The resulting nanocomposites are closer to the electrical percolation threshold. Their conductivities depend strongly on the precise microstructure, which leads to larger variability.

The phase separation model is supported by small-angle X-ray scattering (SAXS) analyses that we performed to assess the average spacing of the gold particle cores. Transmission SAXS on drop-casted films on Kapton with different PVAs (scattering shown in Figure S1 of the Supporting Information) indicated a reduced spacing of the particles upon addition of PVA. Scattering that originates from the gold cores’ structure factor (at *q* = 0.00806 Å^–1^) shifted by up to 0.00842 Å^–1^ in films containing PVA. Average core–core distances of the hybrid particles were determined with Bragg’s equation.^36,37^ We subtracted them from the core–core distance without a PVA of 779.55 Å. The shifts in Figure 3B indicate a reduction of the average core–core distances by 3.3 nm, which is consistent with the removal of PSS from between the hybrid particles. The reduced spacing is caused by the smaller PEDOT alone. The shift increased with the PVA hydrolyzation degree, which is consistent with the formation of hydrogen bonds between PVA and PSS, as illustrated in Figure 3A.

Bulk phase separation of PVA:PSS and PEDOT on hCNP is a likely explanation for the resistivity decrease for PVA_99+%_^89–98^ at 10 vol %. The SAXS results in Figure 3B support this interpretation. Note that the unchanged SPR shift from UV–vis does not contradict our conclusion since PEDOT dominates the dielectric properties of the shell.

### Influence of the PVA Content of the Nanocomposites on Adhesion to PET Substrates

In the following, we analyze the effect of PVA in the nanocomposites on adhesion.

**Figure 4:**
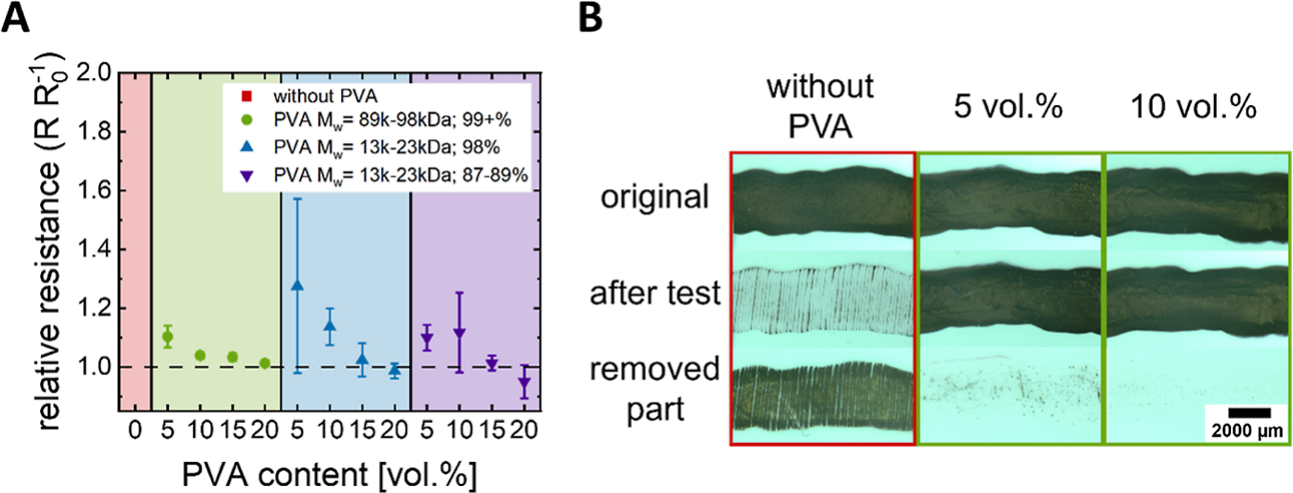
(A) Relative electrical resistances of nanocomposite films after scotch tape tests at different dry volume fractions of PVAs. (B) Optical micrographs were taken before and after adhesion testing of drop-casted samples without PVA and 5 and 10 vol % PVA_99+%_^89–98^.

PVA with a high molecular weight is known to increase adhesion in graphite and graphite/silicon battery electrodes by forming hydrogen bonds.^38^ We tested whether PVA addition increases the adhesion of the hCNP films in a similar fashion. We quantified the effect of PVA on the adhesion of the dried films to PET substrates on nanocomposites with solid PVA contents of 5–20 vol %. Films of pure hybrid particles had poor adhesion (Figure 4) and were easily displaced by external forces. The adhesion of drop-casted samples was assessed using a standard “Scotch tape test” (cf. Characterization section). Film resistances were measured before and after the tests using a two-point setup (Figure 4A). The degradation of the samples was characterized with optical microscopy (Figures 4B and S2 in the Supporting Information).

Films without PVA were entirely removed during the adhesion test, and no measurable conductivity remained after the test. An addition of 5 vol % PVA considerably increased the adhesion of the nanocomposite such that the increase in resistance was below 10 or 28% depending on the PVA type. An addition of 10 vol % PVA reduced the resistance increase to below 4% for PVA_99+%_^89–98^. Scattering of the resistivities after the tests was lower for PVA_99+%_^89–98^ than for any other PVA. This is consistent with the proposed phase separation/interaction mechanism (see above).

We attribute the increased adhesion to the hydrogen bonds between PEDOT:PSS, PVA, and the substrate. Similar hydrogen bonds were described by Zhao et al. in 2010 for the layer-by-layer deposition of graphene oxide and PVA.^39^ Increased hydrolyzation of PVA increases hydrogen bond density and, thus, adhesion.

A further increase of the PVA content reduced the effect of the adhesion test on resistance to almost zero, but at the cost of increased resistivity. In the following, we exclusively used composites with a PVA content of 10 vol % for inkjet-printing as a compromise between resistivity and adhesion.

### Resistivity and Adhesion of Inkjet-Printed Nanocomposites

**Figure 5:**
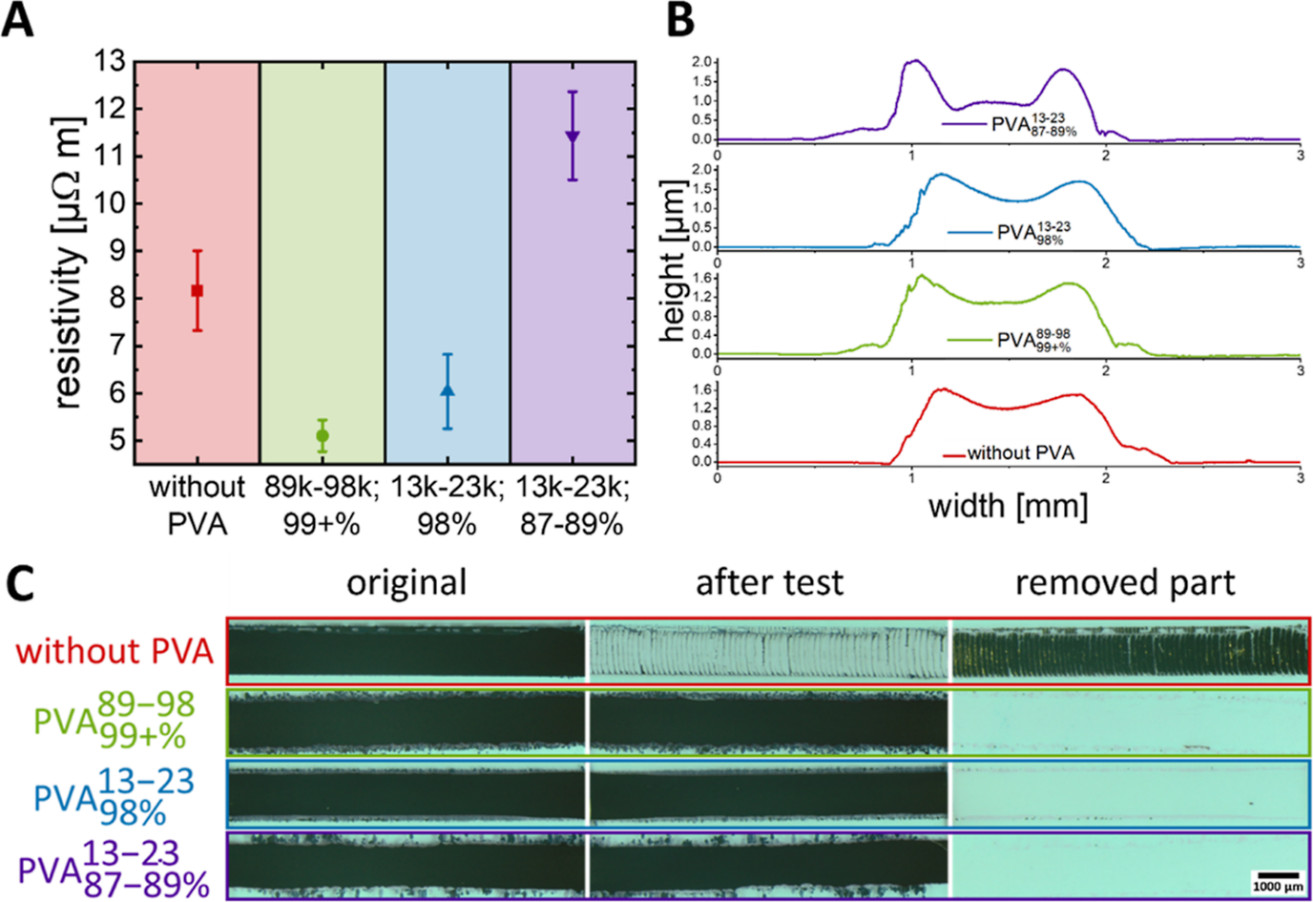
Characterization of inkjet-printed nanocomposite films. (A) Resistivities of films containing 10 vol % PVA. (B) Relative resistance after Scotch tape tests. (C) Micrographs before and after Scotch tape testing (bright: substrate; dark: nanocomposite films).

Inks for inkjet printing were formulated using the results from the previous chapters and printed to obtain dried films containing 10 vol % of PVA_99+%_^89–98^, PVA_98%_^13–23^, or PVA_87–89%_^13–23^. Samples consisting of lines with dimensions of 1 × 10 mm on PET had resistivities of 8.2 μΩ m (without PVA), 5.1 μΩ m (PVA_99+%_^89–98^), 6.0 μΩ m (PVA_98%_^13–23^), and 11.4 μΩ m (PVA_87–89%_^13–23^) (Figure 5A).

Processing can affect the (micro)structure of the films and thus their conductivity. Figure 5B shows height profiles of inkjet-printed samples with edges that are 1.30 μm thicker than the center with PVA_87–89+%_^13–23^, an effect that is considerably stronger than that in drop casting.

PVA molecular weight and degree of hydrolysis had stronger effects on the conductivities of inkjet-printed samples than on drop-casted samples. Inkjet-printed samples with PVA_99+%_^89–98^ had 63% of the conductivity of pure hCNP layers, and those with PVA_98%_^13–23^ had 74% compared to 92 and 144% for drop-casted samples. The viscosities of the inks were too similar (1.6–2.0 Pa s at 50.1 s^–1^, data not shown here) to explain this difference. It seems likely that the inkjet printing process with its small droplets changes the demixing process and leads to less conductive microstructures. For example, it seems conceivable that the drying droplets enrich PVA in their shells, which later form insulating barriers that are not present in drop casting.

There was no noticeable difference in adhesion between the drop-cast (Figure 4B) and inkjet-printed samples (Figure 5C). Samples without PVA were almost completely removed by the Scotch tape test, whereas all samples with 10 vol % PVA were stable (Figure 5C).

A content of 10 vol % of all used PVAs led to an inkjet-printable nanocomposite with improved adhesion and comparable resistances. In the following, we study the effect of PVA on the bendability and tensile strength of inkjet-printed films.

### Deformation Stability of Inkjet-Printed Hybrid Nanocomposites

Printed particle films on flexible and stretchable substrates can sustain limited deformation without failing,^40^ but resistivity generally increases. In the following, we report the effect of PVA types in hCNP–PVA composites on the resistances of bent and stretched films.

**Figure 6:**
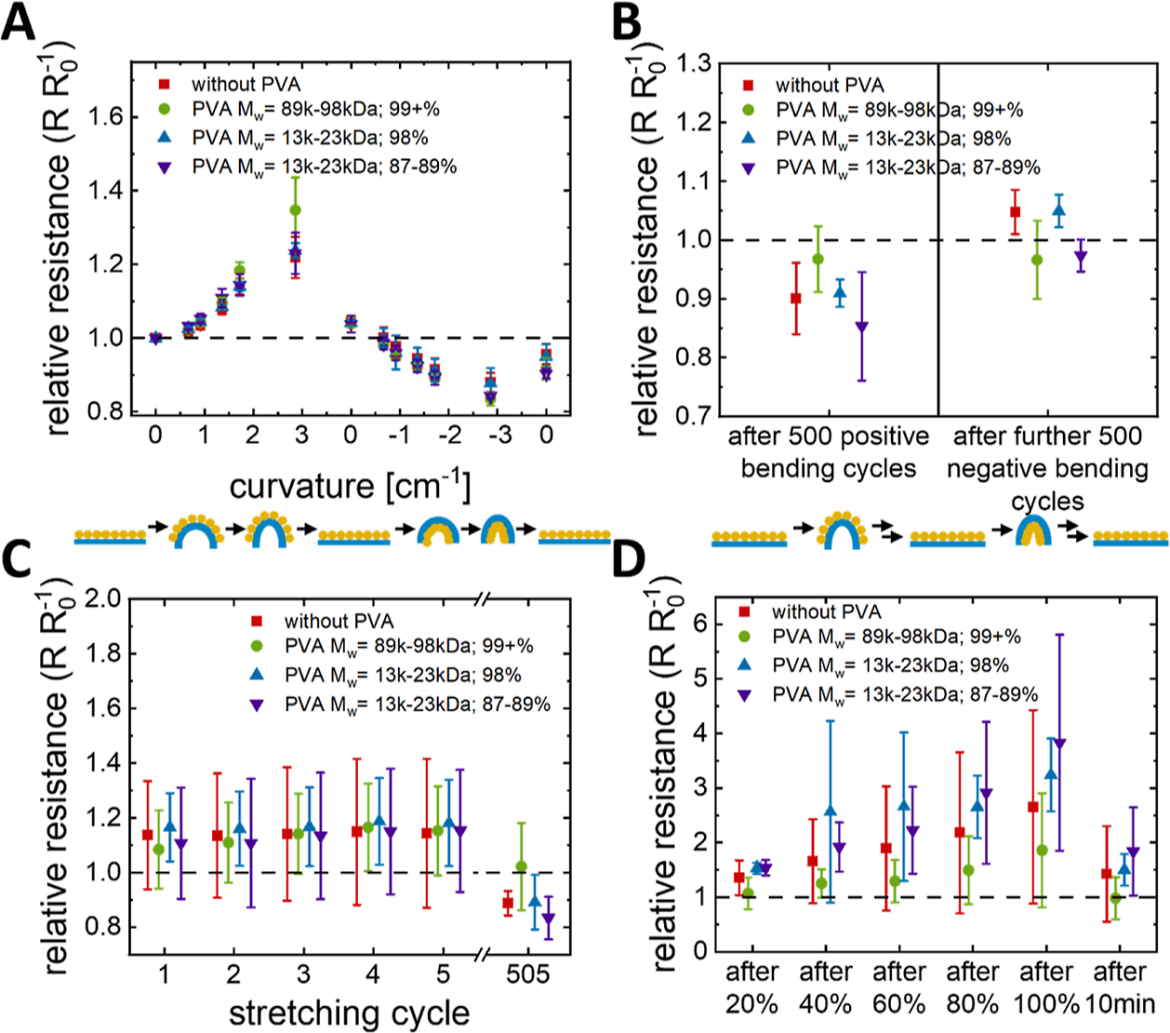
Mechanical stability of inkjet-printed nanocomposites on PET (bending) and PU (stretching) substrates. The plots show relative resistances (A) after bending the sample once to the given curvature, (B) after 500 bending cycles to 3.1 cm^–1^, (C) after 5 stretching cycles, and after 10 min relaxation after a further 500 stretching cycles to a strain of 10% at 2% s^–1^, (D) after stretching to 20, 40, 60, 80, and 100% strain and after 10 min relaxation.

Lines printed on PET were electrically contacted with silver paste, and the resistances were determined at different bending radii (Figure 6A). Positive curvatures (where the conductive layer was stretched, see Figure 6A bottom left) increased resistance: the highest curvature of 2.86 cm^–1^ by a factor of 1.3 for 10 vol % PVA_99+%_^89–98^ and 1.2 for all other samples. Releasing the curvature removed the strain, and the resistance immediately dropped to its original value. The resistance increases are readily explained by the tensile strain that induced stresses, increased particle–particle distances, and possibly initiated microcrack formation. Majee et al. showed that layers of inkjet-printed zinc nanoparticles formed cracks upon bending with <3 mm radius of curvature, which increased sheet resistance.^40^

Negative curvatures (where the conductive layer was compressed) reduced resistances by a factor of 0.8–0.9 at −2.86 cm^–1^, with the change reversible upon release. Compression of the particle packing reduced interfacial spacing and formed additional conductive pathways or reduced contact resistance in the existing paths. Wang et al. 2023 reported similar observations in porous composites of PDMS and carbon black that they prepared for strain sensors.^41^

We found no change in the electrical response to bending upon the addition of PVA, except for PVA_99+%_^89–98^. This is again consistent with the phase separation picture presented above. Previous reports indicate that PVA_99+%_^89–98^ is less flexible than other PVA types,^35^ which may explain the exception. Cracks that form in the PVA phase and propagate into the conductive phase are expected to reduce the conductivity. Particle mobility may be an additional factor: hCNPs retain a certain mobility in the dry film, in contrast to sintered particles.^42^ This mobility probably aids deformation and stabilizes the resistance during bending. It is conceivable that PVA reduces the mobility of the hCNPs in the PVA–hCNPs composite because the PVA phase stabilizes the arrangement.

We investigated the stability against cyclical bending, too. The resistances during 500 bends to a positive curvature of 3.1 cm^–1^ were measured in situ, followed by the same experiment but to −3.1 cm^–1^ (Figure 6B). The final resistance increases were always below 10% (related to the resistance before the entire cyclical bending), excluding major crack formation and suggesting a microscale reconfiguration. This reversibility indicates that percolating networks of hCNPs that were disrupted during bending are reestablished upon strain release. The effects of PVA addition were insignificant for all of the cases.

In summary, PVA did not affect the stability of the printed layers against bending when compared to the much softer layers composed of pure hCNP. This is consistent with the emergence of an elastic PVA matrix that is reversibly deformable but does not affect the microscopic deformation mechanisms of the conductive phase.

Uniaxial strain is a practically relevant deformation for stretchable electronics and an important reference case for the analysis of deformation mechanisms. We performed tensile testing on lines that were printed on PU. The specimens were clamped in the tensile test machine and strained, and the resistances were measured. Stretching tests were performed by straining five times to 10% in 2% s^–1^ (Figure 6C), with concurrent resistance investigations to exclude initial strong changes in resistance. The relative changes of resistances between relaxed states (measured 20–30 s after relaxing the force) were in the range of 0.9–1.4 and did not change significantly during the first five cycles. The effect of PVA addition on comparison of resistances before and after stretching was insignificant for all cases. During stretching, the conductivities of the samples with PVA decreased rapidly and temporarily dropped to zero.

Cyclical testing with 500 cycles of up to 10% strain at a rate of 2% s^–1^ was performed to test the stability with concurrent resistance investigations. The relative resistances as measured directly after 500 cycles are between 2.5 and 4.4 and between 0.8 and 1.2 approximately 10 min after the last cycle (Figure 6C), compared to the resistances after 5 cycles. The origin of the reduction are slow relaxation processes.

Stretching the same samples to progressively larger strains (20, 40, 60, 80, and 100%) led to increasingly larger resistance changes, as measured 20–30 s after relaxation (Figure 6D). Relative resistances increased up to 5.8 but fell to 2.6 or below 10 min after the last stretching. Thermoplastic polyurethanes slowly relax after release, explaining the slow return.^43^ Straining to 100% led to partial plastic deformation and buckling after the force was released, which caused considerable differences between the individual samples. Overall, samples with PVA_99+%_^89–98^ degraded less than composites with PVA_98%_^13–23^ and PVA_87–89%_^13–23^ (Figure 6D). The tensile strength of PVA increases with hydrolysis and molecular weight;^35^ the observed differences probably reflect the different mechanism properties of the PVA phase.

## Conclusions

We created drop-cast/inkjet-printed nanocomposites with small volume fractions of PVA to stabilize sinter-free, PEDOT:PSS-coated gold nanoparticles. The resulting materials were highly conductive when using highly hydrolyzed PVA with large molecular weights at volume fractions up to 10 vol %. The conductivity of some nanocomposites exceeded that of pure hCNP layers.

All tested PVA types increased adhesion in Scotch tape tests, some so much that no damage was detected but at the cost of limited electrical conductivity. Nanocomposites of 10 vol % PVA are multiple stretchable to 10% (with a reversible reduction in resistance to 0.8–1.2) and did not influence bendability.

Our results are consistent with the existence of a PVA phase or network that spans the composite but does not interrupt the percolating hCNP network at PVA volume fractions below 15 vol %. The mechanical properties of the composites are enhanced by the PVA phase depending on their molecular weight and hydrolysis-dependent properties. Hydrogen bonds between PVA and PEDOT:PSS are likely to form and cause the formation of a separate PSS phase.

Current studies focus on the structure of the PVA network at nano- and micrometer scales. The detailed structure and their stability, e.g., against large humidities, will be reported in a forthcoming paper.

## Experimental Section

### Materials

Tetrachloric(III) acid trihydrate (HAuCl_4_·3H_2_O, ≥49.0% Au, Acros Organics, USA), hexadecyltrimethylammonium bromide (CTAB, >99%, Sigma-Aldrich, Germany), sodium oleate (Na-oleat, >82% fatty acids, Sigma-Aldrich, Germany), silver nitrate (AgNO_3_, ≥99.0%, Sigma-Aldrich, Germany), sodium borohydrate (NaBH_4_, >98.0%, Sigma-Aldrich, Germany), and L-ascorbic acid (≥98%, Sigma-Aldrich, Germany) were used for the synthesis of CTAB-stabilized gold nanoparticles. Commercial dispersions of PEDOT:PSS were used to form the conductive ligand shell. We applied Clevios P or Clevios P10 (1.3 wt %, Heraeus, Germany) for standard and Clevios P10 for inkjet-printable nanocomposites (see discussion below). Clevios P and Clevios P10 have identical specifications (solid content 1.2–1.4%, surface resistivity ≤1000 kΩ q^–1^) and were provided by the same suppliers (Heraeus, Germany), but with different raw-material suppliers.

Nanocomposites were formed by adding PVA with *M*_w_ 89,000–98,000, 99+% hydrolyzed, PVA *M*_w_ 13,000–23,000, 98% hydrolyzed, or PVA *M*_w_ 13,000–23,000, 87–89% hydrolyzed (all from Sigma-Aldrich, Germany). We denote the polymers using PVA_*x*%_^*y*–*z*^ in the following, where *x* represents the hydrolyzation degree in percent and *y*–*z* the PVA molecular weight range in kDa.

All chemicals were used without further purification. Purified water (double reverse osmosis) was used in particle synthesis and to dissolve PVA. The water was purified with a Mili-Q Advantage system (Merck, Darmstadt, Germany).

Nanocomposite films and films without PVA were deposited by drop-casting on glass slides (microscopy soda-lime glass slides, Carl Roth GmbH, Germany) or poly(ethylene terephthalate) (PET) foil (Melinex ST504/125 μm, Pütz GmbH, Germany). Inkjet printing was tested on PET and PU foils (Platilon U9122 natural 150 μm, Epurex Films GmbH, Germany). Double-sided adhesive copper tape (Plano GmbH, Germany) and silver paste (ACHESON silver DAG 1415, Plano GmbH, Germany) were used to establish electrical contacts between the thin conductive films and the electrical leads. The substrates were cleaned with ethanol (99%, Brenntag, Germany) and isopropyl alcohol (99%, Brenntag, Germany).

### Synthesis of hCNPs

Hybrid nanoparticles with gold cores and PEDOT:PSS shells were synthesized using a protocol adapted from Escudero et al.^13^ In this work, we used two different batches of hybrid gold nanoparticles: batch 1 had Clevios P as a conductive ligand and was used for all drop-casted samples for material development. Batch 2 had Clevios P10 and was used for all inkjet-printed samples.

In a typical synthesis, the seed solution was prepared by mixing 90 mL of a 0.2 molar CTAB solution with 90 mL of a 0.5 molar gold acid solution at 40 °C while stirring; 10 mL of a 6.9 molar sodium borohydride solution was added. After being stirred for 1–2 min, the solution was stored at 40 °C for 30 min.

A 10 L flat ground flange reactor vessel was filled with 43.6 g of sodium oleate, 351 g of CTAB, and 7900 mL of Milli-Q water, stirred at 45 °C until complete dissolution, and then kept under stirring at 40 °C during the entire synthesis. Using two beakers, 10 mL of a 0.085 molar silver nitrate solution and then 85 mL of a 0.436 molar gold acid solution were added rapidly. Visible solids formed and dissolved after approximately 1 h. The seed solution was added rapidly when no solids were visible in the solution.

25 mL portion of a 0.652 molar ascorbic acid solution was added with a syringe driver at a rate of 0.6 mL h^–1^. The resulting, CTAB-stabilized gold nanoparticles were concentrated and purified by centrifuging (1 h with 7270 rcf in 1.5 L beakers and 45 min with 4010 rcf in 50 mL falcon tubes) and redispersing several times. After the last centrifugation step, the particles were redispersed in a 5 mM CTAB solution with a total volume of approximately 90 mL (distributed over two falcon tubes)

For ligand exchange, 405 g of Clevios P or Clevios P10 was mixed with 7790 mL of Milli-Q water in the 10 L reactor, and 110 mL of CTAB-stabilized gold nanoparticles was added. Another 100 mL of Milli-Q water was used to rinse the vessel of the CTAB-stabilized particles, and the mixture was added to the reactor. The mixture was stirred overnight to obtain PEDOT:PSS-stabilized gold nanoparticles. They were concentrated by centrifugation (1 h with 7270 rcf in 1.5 L beakers and 4650 rcf in 50 mL falcon tubes), and the excess PEDOT:PSS was removed. The dispersion was filtered, washed until the supernatant was light pink, and concentrated to approximately 27 mL. This produced hCNPs dispersion (hybrid ink) with more than 130 mg mL^–1^ gold content.

### Nanocomposite Preparation

For the preparation of 5 wt % PVA stock solutions, 750 mg of the respective PVA types and 14.25 g of Milli-Q water were mixed and ultrasonicated for 10 s. The mixtures were heated to 85 °C for 1 h while stirring, the hot plate was turned off, and the dispersion cooled to room temperature under stirring (45 min).

The hybrid ink was diluted to 130 mg mL^–1^ gold content and mixed with the PVA stock solutions to obtain composites with PVA contents of 5, 10, 15, 20, 25, and 30 vol % PVA.

### Film Deposition

Substrates were cleaned for 15 min with a 1:1 solution of 2-propanol and ethanol in an ultrasonic bath (Elmasonic S100H, 37 kHz, effective ultrasonic power 150 W) and then plasma-treated during 10 min in an argon–hydrogen plasma (Pico, Diener electronic, 100 W).

### Drop Casting and Inkjet Printing

Substrates for drop casting were activated with oxygen plasma (Pico, Diener electronic, 100 W). Lines were produced by casting from an Eppendorf pipette. 2 μL of ink was used to cast four lines each for the calculation of resistivity and adhesion experiments.

Substrates (PET foil and PU foil) for inkjet printing were activated with oxygen plasma (PlasmaFlecto30, plasma technology, 200 W). Lines were prepared by using the PixDro LP50 printer from MeyerBurger (today SÜSS MicroTec) and the DIMATIX DMC11610 cartridges. Customized voltage profiles (trapezial waveform) with a maximum voltage of 31 V (at droplet ejection) and a minimum voltage of 4 V (for chamber filling) were used to drive the piezo print heads; waveforms were adapted for each printing. We inkjet-printed electrode areas of 1 mm × 10 mm to measure conductivities and adhesion and lines with 1 mm × 15–20 mm during bending and stretching with a resolution of 3000 dpi and a printing speed of 150 mm s^–1^.

All drop-casted and inkjet-printed samples were dried for 7 days at 21.8 °C and a relative humidity of 30–60% before mechanical and electrical characterization. Note that drying times affected resistivities depending on the PVA type and volume fraction (see Figure S3 in the Supporting Information).

### Characterization

The density of PVA was determined with a gas pycnometer (AccuPyc 1330, Micromeritics, Germany) averaging over 250 measurements for each powder. The results were used to calculate the volume fractions reported here. The viscosities of hCNPs–PVA solutions were determined with a rheometer after an equilibration time of 30 s at shear rates between 0.1 and 100 s^–1^ at 20 °C (MCR302e, Anton Paar, Germany). The gap of the plate-cone assembly (50 mm diameter, angle 1°) was 98 μm. The optical transmissions of the nanoparticle dispersions were characterized with a UV–vis/NIR spectrometer (Carry 5000, Agilent, USA) in the range 350–800 nm to analyze the colloidal stability of the gold particles, and the gold concentrations were determined by ICP-OES (Ultima2, Horiba Scientific, Japan).

Electrical conductivities were determined using voltage sweeps in the range of −25 to +25 mV with concurrent current measurements that provided current–voltage curves. We used two- or four-point probes connected to a 2450 SourceMeter (Keithley instruments, USA); the data were analyzed by the slope of voltage–current curves. Two-point-probe measurements were conducted with samples connected by using silver paste at each end; four-point-probe measurements were performed using gold-coated copper beryllium electrodes at a spacing of 1 mm in the center of the sample. The thicknesses of the films were measured with a confocal microscope (MarSurf CM explorer, Mahr GmbH, Germany) and used to calculate the resistivities ρ of the nanocomposites as
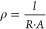
1where *l* is the electrode spacing, *R* is the measured resistance, and *A* is the cross-sectional area.

The adhesion test mimicked the scotch tape test published in the technical note IPC-TM-650 TM2.4.1E by IPC International, a trade association that defines industry standards. Scotch tapes (Scotch Crystal Tape, 3M) were pressed on the drop-casted/printed line and removed by a rapid movement at an angle of about 180° less than 1 min after attachment. Going beyond the standard, optical micrographs were taken with an SZX16 microscope (Olympus, Japan) to analyze the loss of material during adhesion tests.

We monitored the resistance of the samples during bending around rolled rim glass vials with different diameters (3.0, 2.2, 1.5, 1.2, and 0.7 cm) with a 2450 SourceMeter (Keithley instruments, USA). For multiple bending tests, the samples were bent 500 times to one side (3.1 cm^–1^) and 500 times to the other side (−3.1 cm^–1^) during the measurement of the resistance with a multimeter (DAQ6510, Keithley Instruments, USA).

We analyzed the resistance during stretching with a tensile test machine (Zwick 1446, ZwickRoell GmbH, Germany) and a multimeter (DAQ6510, Keithley Instruments, USA). The samples were stretched to different strains (10, 20, 40, 60, 80, and 100%) and released to their original state, and the final resistance was determined.

A Xeuss 2.0 instrument (Xenocs SA, France) was used for the SAXS measurements. The inks were drop-casted on Kapton and measured with a sample-detector distance of 2.512 m, in the range of 0.004–0.224 Å, for 3600 s and an X-ray with a wavelength of 1.54 Å.

## Supporting Information Available

Small-angle X-ray scattering measurements of drop-casted samples on Kapton, optical microscopy of adhesion tests with different PVA types and contents, and effect of drying time, type, and PVA content on the resistivity of drop-casted samples (PDF)

## Abbreviations

PVA: poly(vinyl alcohol)
PEDOT:PSS: poly(3,4-ethylenedioxythiophene) polystyrenesulfonate
hCNP(s): hybrid conductive nanoparticle(s)
SAXS: small-angle X-ray scattering

## References

[ref1] Choi S.; Lee S.; Lee B.; Yoon J.; Lee C. Y.; Kim T.; Hong Y. Crack-Inducing Strain Sensor Array Using Inkjet-Printed Silver Thin Film for Underplate and Off-Centered Force Sensing Applications. ACS Appl. Mater. Interfaces 2023, 15 (3), 4487–4494. 10.1021/acsami.2c19372. 36642889

[ref2] Tursunniyaz M.; Agarwal V.; Meredith A.; Andrews J. Hybrid Nanomaterial Inks for Printed Resistive Temperature Sensors with Tunable Properties to Maximize Sensitivity. Nanoscale 2023, 15 (1), 162–170. 10.1039/D2NR04005K. 36478149

[ref3] Hamoy F. N. P.; Romero D. G.; Macaraig L. C. D.; Enriquez E. P. Inkjet Printing of UHF RFID Antennas Using Silver and Gold Inks. Key Eng. Mater. 2022, 913, 35–44. 10.4028/p-5v0h1p.

[ref4] Wang F.; Mao P.; He H. Dispensing of High Concentration Ag Nano-Particles Ink for Ultra-Low Resistivity Paper-Based Writing Electronics. Sci. Rep. 2016, 6 (1), 21398 10.1038/srep21398. 26883558 PMC4756288

[ref5] Jung H. C.; Cho S. H.; Joung J. W.; Oh Y. S. Studies on Inkjet-Printed Conducting Lines for Electronic Devices. J. Electron. Mater. 2007, 36 (9), 1211–1218. 10.1007/s11664-007-0194-5.

[ref6] Skarżyński K.; Krzemiński J.; Jakubowska M.; Słoma M. Highly Conductive Electronics Circuits from Aerosol Jet Printed Silver Inks. Sci. Rep. 2021, 11 (1), 18141 10.1038/s41598-021-97312-5. 34518558 PMC8437949

[ref7] Kuang M.; Wang L.; Song Y. Controllable Printing Droplets for High-Resolution Patterns. Adv. Mater. 2014, 26 (40), 6950–6958. 10.1002/adma.201305416. 24687946

[ref8] Kim D.; Moon J. Highly Conductive Ink Jet Printed Films of Nanosilver Particles for Printable Electronics. Electrochem. Solid-State Lett. 2005, 8 (11), 130–133. 10.1149/1.2073670.

[ref9] Choi Y.; Seong K.-D.; Piao Y. Metal-Organic Decomposition Ink for Printed Electronics. Adv. Mater. Interfaces 2019, 6 (20), 1901002 10.1002/admi.201901002. 24512011

[ref10] Kwak J.; Bae W. K.; Zorn M.; Woo H.; Yoon H.; Lim J.; Kang S. W.; Weber S.; Butt H. J.; Zentel R.; Lee S.; Char K.; Lee C. Characterization of Quantum Dot/Conducting Polymer Hybrid Films and Their Application to Light-Emitting Diodes. Adv. Mater. 2009, 21 (48), 5022–5026. 10.1002/adma.200902072. 25378234

[ref11] Zorn M.; Bae W. K.; Kwak J.; Lee H.; Lee C.; Zentel R.; Char K. Quantum Dot-Block Copolymer Hybrids with Improved Properties and Their Application to Quantum Dot Light-Emitting Devices. ACS Nano 2009, 3 (5), 1063–1068. 10.1021/nn800790s. 19845366

[ref12] Drzic J.; Escudero A.; González-García L.; Kraus T. Sacrificial Ligand Route to Hybrid Polythiophene-Silver Nanoparticles for Sinter-Free Conductive Inks. Inorg. Chem. Front. 2023, 10 (5), 1552–1560. 10.1039/D2QI02722D.

[ref13] Escudero A.; Gonzalez-Garcia L.; Strahl R.; Kang D. J.; Drzic J.; Kraus T. Large-Scale Synthesis of Hybrid Conductive Polymer-Gold Nanoparticles Using “Sacrificial” Weakly Binding Ligands for Printing Electronics. Inorg. Chem. 2021, 60 (22), 17103–17113. 10.1021/acs.inorgchem.1c02350. 34735769

[ref14] Reiser B.; González-García L.; Kanelidis I.; Maurer J. H. M.; Kraus T. Gold Nanorods with Conjugated Polymer Ligands: Sintering-Free Conductive Inks for Printed Electronics. Chem. Sci. 2016, 7 (7), 4190–4196. 10.1039/C6SC00142D. 30155064 PMC6014069

[ref15] Luo X. M.; Zhang B.; Zhang G. P. Fatigue of Metals at Nanoscale: Metal Thin Films and Conductive Interconnects for Flexible Device Application. Nano Mater. Sci. 2019, 1 (3), 198–207. 10.1016/j.nanoms.2019.02.003.

[ref16] Luo X. M.; Zhang G. P. Grain Boundary Instability Dependent Fatigue Damage Behavior in Nanoscale Gold Films on Flexible Substrates. Mater. Sci. Eng., A 2017, 702, 81–86. 10.1016/j.msea.2017.07.006.

[ref17] Meyers M. A.; Mishra A.; Benson D. J. Mechanical Properties of Nanocrystalline Materials. Prog. Mater. Sci. 2006, 51 (4), 427–556. 10.1016/j.pmatsci.2005.08.003.

[ref18] Bora C.; Gogoi P.; Baglari S.; Dolui S. K. Preparation of Polyester Resin/Graphene Oxide Nanocomposite with Improved Mechanical Strength. J. Appl. Polym. Sci. 2013, 129 (6), 3432–3438. 10.1002/app.39068.

[ref19] Kumar K.; Ghosh P. K.; Kumar A. Improving Mechanical and Thermal Properties of TiO2-Epoxy Nanocomposite. Composites, Part B 2016, 97, 353–360. 10.1016/j.compositesb.2016.04.080.

[ref20] Crosby A. J.; Lee J.-Y. Polymer Nanocomposites: The “Nano” Effect on Mechanical Properties. Polym. Rev. 2007, 47 (2), 217–229. 10.1080/15583720701271278.

[ref21] Khalilnezhad P.; Sajjadi S. A.; Zebarjad S. M. Effect of Nanodiamond Surface Functionalization Using Oleylamine on the Scratch Behavior of Polyacrylic/Nanodiamond Nanocomposite. Diamond Relat. Mater. 2014, 45, 7–11. 10.1016/j.diamond.2014.03.002.

[ref22] Rafiee M. A.; Rafiee J.; Wang Z.; Song H.; Yu Z. Z.; Koratkar N. Enhanced Mechanical Properties of Nanocomposites at Low Graphene Content. ACS Nano 2009, 3 (12), 3884–3890. 10.1021/nn9010472. 19957928

[ref23] Chantarachindawong R.; Luangtip W.; Chindaudom P.; Osotchan T.; Srikhirin T. Development of the Scratch Resistance on Acrylic Sheet with Basic Colloidal Silica (SiO 2)-Methyltrimethoxysilane (MTMS) Nanocomposite Films by Sol-Gel Technique. Can. J. Chem. Eng. 2012, 90 (4), 888–896. 10.1002/cjce.21631.

[ref24] Rivière L.; Lonjon A.; Dantras E.; Lacabanne C.; Olivier P.; Gleizes N. R. Silver Fillers Aspect Ratio Influence on Electrical and Thermal Conductivity in PEEK/Ag Nanocomposites. Eur. Polym. J. 2016, 85, 115–125. 10.1016/j.eurpolymj.2016.08.003.

[ref25] Kim E. J.; Yeum J. H.; Choi J. H. Effects of Polymeric Stabilizers on the Synthesis of Gold Nanoparticles. J. Mater. Sci. Technol. 2014, 30 (2), 107–111. 10.1016/j.jmst.2013.11.012.

[ref26] Shi H.; Xu N.; Zhao D.; Xu B. Q. Immobilized PVA-Stabilized Gold Nanoparticles on Silica Show an Unusual Selectivity in the Hydrogenation of Cinnamaldehyde. Catal. Commun. 2008, 9 (10), 1949–1954. 10.1016/j.catcom.2008.03.025.

[ref27] Amendola V.; Pilot R.; Frasconi M.; Maragò O. M.; Iatì M. A. Surface Plasmon Resonance in Gold Nanoparticles: A Review. J. Phys.: Condens. Matter 2017, 29 (20), 203002 10.1088/1361-648X/aa60f3. 28426435

[ref28] Zook J. M.; Rastogi V.; MacCuspie R. I.; Keene A. M.; Fagan J. Measuring Agglomerate Size Distribution and Dependence of Localized Surface Plasmon Resonance Absorbance on Gold Nanoparticle Agglomerate Size Using Analytical Ultracentrifugation. ACS Nano 2011, 5 (10), 8070–8079. 10.1021/nn202645b. 21888410

[ref29] Wang G.; Wang C.; Tang C.; Zhang F.; Sun T.; Yu X. Two-Stage Electrical Percolation of Metal Nanoparticle-Polymer Nanocomposites. J. Phys. Chem. C 2018, 122 (15), 8614–8620. 10.1021/acs.jpcc.8b01079.

[ref30] Chen Q.; Sochor B.; Chumakov A.; Betker M.; Ulrich N. M.; Toimil-Molares M. E.; Gordeyeva K.; Söderberg L. D.; Roth S. V. Cellulose-Reinforced Programmable and Stretch-Healable Actuators for Smart Packaging. Adv. Funct. Mater. 2022, 32 (49), 2208074 10.1002/adfm.202208074.

[ref31] Wang X.-Y.; Feng G.-Y.; Li M.-J.; Ge M.-Q. Effect of PEDOT:PSS Content on Structure and Properties of PEDOT:PSS/Poly(Vinyl Alcohol) Composite Fiber. Polym. Bull. 2019, 76 (4), 2097–2111. 10.1007/s00289-018-2459-y.

[ref32] Fallahzadeh A.; Saghaei J.; Yousefi M. H. Effect of Alcohol Vapor Treatment on Electrical and Optical Properties of Poly(3,4-Ethylene Dioxythiophene):Poly(Styrene Sulfonate) Films for Indium Tin Oxide-Free Organic Light-Emitting Diodes. Appl. Surf. Sci. 2014, 320, 895–900. 10.1016/j.apsusc.2014.09.143.

[ref33] Zhang P.; Reiser B.; González-García L.; Beck S.; Drzic J.; Kraus T. Drying of Electrically Conductive Hybrid Polymer-Gold Nanorods Studied with: In Situ Microbeam GISAXS. Nanoscale 2019, 11 (14), 6538–6543. 10.1039/C8NR09872G. 30907898

[ref34] Hassan C. M.; Peppas N. A. Structure and Applications of Poly(vinyl alcohol) Hydrogels Produced by Conventional Crosslinking or by Freezing/Thawing Methods. Biopolymers PVA Hydrogels, Anionic Polymerisation Nanocomposites; Springer, 2000; Vol. 153, pp 37–65.

[ref35] Nawaz A.; Hümmelgen I. A. Poly(Vinyl Alcohol) Gate Dielectric in Organic Field-Effect Transistors. J. Mater. Sci.: Mater. Electron. 2019, 30, 5299–5326. 10.1007/s10854-019-00873-5.

[ref36] Groppe P.; Reichstein J.; Carl S.; Cuadrado Collados C.; Niebuur B. J.; Zhang K.; Apeleo Zubiri B.; Libuda J.; Kraus T.; Retzer T.; Thommes M.; Spiecker E.; Wintzheimer S.; Mandel K. Catalyst Supraparticles: Tuning the Structure of Spray-Dried Pt/SiO2 Supraparticles via Salt-Based Colloidal Manipulation to Control Their Catalytic Performance. Small 2024, 2310813 10.1002/smll.202310813. 38700050

[ref37] Wang X.; Zhang Y.; Huang J.; Tian C.; Xia M.; Liu L.; Li Z.; Cao J.; Gui S.; Chu X. A Novel Phytantriol-Based Lyotropic Liquid Crystalline Gel for Efficient Ophthalmic Delivery of Pilocarpine Nitrate. AAPS PharmSciTech 2019, 20 (1), 32 10.1208/s12249-018-1248-0. 30603986

[ref38] Park H. K.; Kong B. S.; Oh E. S. Effect of High Adhesive Polyvinyl Alcohol Binder on the Anodes of Lithium Ion Batteries. Electrochem. Commun. 2011, 13 (10), 1051–1053. 10.1016/j.elecom.2011.06.034.

[ref39] Zhao X.; Zhang Q.; Hao Y.; Li Y.; Fang Y.; Chen D. Alternate Multilayer Films of Poly(Vinyl Alcohol) and Exfoliated Graphene Oxide Fabricated via a Facial Layer-by-Layer Assembly. Macromolecules 2010, 43 (22), 9411–9416. 10.1021/ma101456y.

[ref40] Majee S.; Karlsson M. C. F.; Wojcik P. J.; Sawatdee A.; Mulla M. Y.; Alvi N. u. H.; Dyreklev P.; Beni V.; Nilsson D. Low Temperature Chemical Sintering of Inkjet-Printed Zn Nanoparticles for Highly Conductive Flexible Electronic Components. npj Flexible Electron. 2021, 5 (1), 14 10.1038/s41528-021-00111-1.

[ref41] Wang Y. F.; Yoshida A.; Takeda Y.; Sekine T.; Kumaki D.; Tokito S. Printed Directional Bending Sensor with High Sensitivity and Low Hysteresis for Human Motion Detection and Soft Robotic Perception. Sensors 2023, 23 (11), 5041 10.3390/s23115041. 37299768 PMC10255501

[ref42] Kang D. J.; Jüttke Y.; González-García L.; Escudero A.; Haft M.; Kraus T. Reversible Conductive Inkjet Printing of Healable and Recyclable Electrodes on Cardboard and Paper. Small 2020, 16 (25), 2000928 10.1002/smll.202000928. 32462772

[ref43] Dutta J.; Naskar K. Investigation of Morphology, Mechanical, Dynamic Mechanical and Thermal Behaviour of Blends Based on Ethylene Vinyl Acetate (EVA) and Thermoplastic Polyurethane (TPU). RSC Adv. 2014, 4 (105), 60831–60841. 10.1039/C4RA07823C.

