# Supplementary material for: Mechanically Robust, Inkjet-Printable Polymer Nanocomposites with Hybrid Gold Nanoparticles and Metal-like Conductivity
Source: ACS Appl Mater Interfaces. 2024 Jun 11;16(24):31576–85. doi: 10.1021/acsami.4c04692 (PMC11195551; doi:10.1021/acsami.4c04692)
Supplement: Supplementary file 1 — am4c04692_si_001.pdf [file am4c04692_si_001.pdf]

Supporting Information

Mechanically Robust, Inkjet-Printable Polymer Nanocomposites with Hybrid Gold Nanoparticles and Metal-like Conductivity

Michael A. H. Klos,[†,‡] *Lola González-García,*[†,§] *and Tobias Kraus*[†,‡*]

†INM – Leibniz Institute for New Materials, Campus D2 2, 66123 Saarbrücken, Germany

‡Saarland University, Colloid and Interface Chemistry, Campus D2 2, 66123 Saarbrücken, Germany

§Saarland University, Department of Materials Science and Engineering, Campus D2 2, 66123 Saarbrücken, Germany

* Corresponding Author

Email: tobias.kraus@leibniz-inm.de

The supporting information includes Small-Angle X-Ray Scattering of drop-casted samples on Kapton (Figure S1), optical microscopy images before and after adhesion tests (Figure S2), and more detailed insight into the effect of drying time, PVA content, and types of PVA on resistivity (Figure S3):

1. Small-Angle X-Ray Scattering of drop-casted samples on Kapton.

2. Optical microscopy of adhesion tests with different type, and PVA contents.

3. Effect of drying time, type, and PVA content on the resistivity of drop-casted samples.

1. Small-Angle X-ray Scattering of drop-casted samples on Kapton.

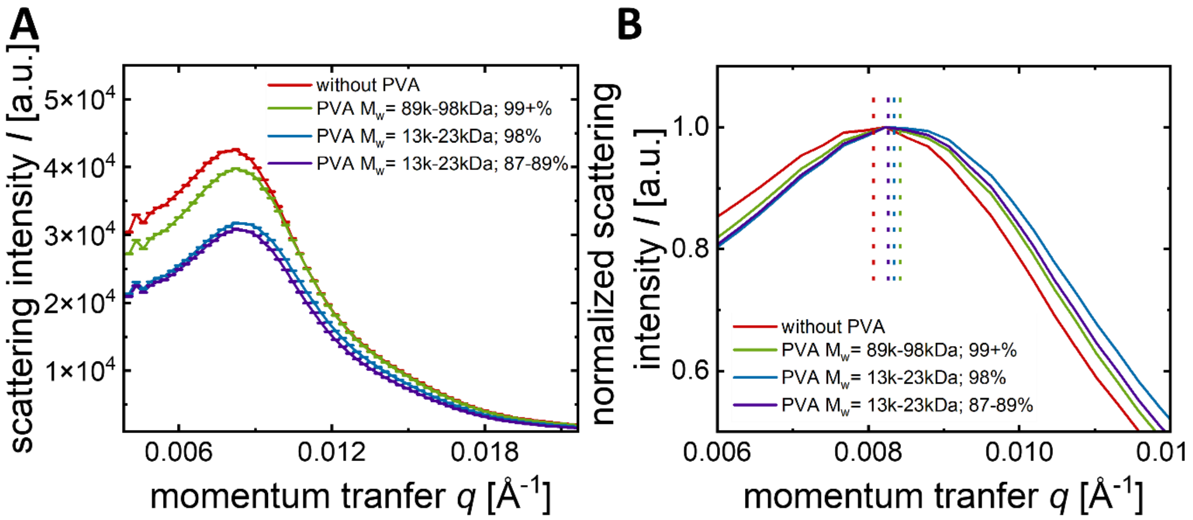

Figure S1: Small-Angle X-ray Scattering measurements of drop-casted inks with and without PVA on Kapton. **A** The scattering intensities of four different samples with and without PVA. **B** Normalized scattering intensity and marked maxima of the four different samples.

2. Optical microscopy of adhesion tests with different PVA types and contents.

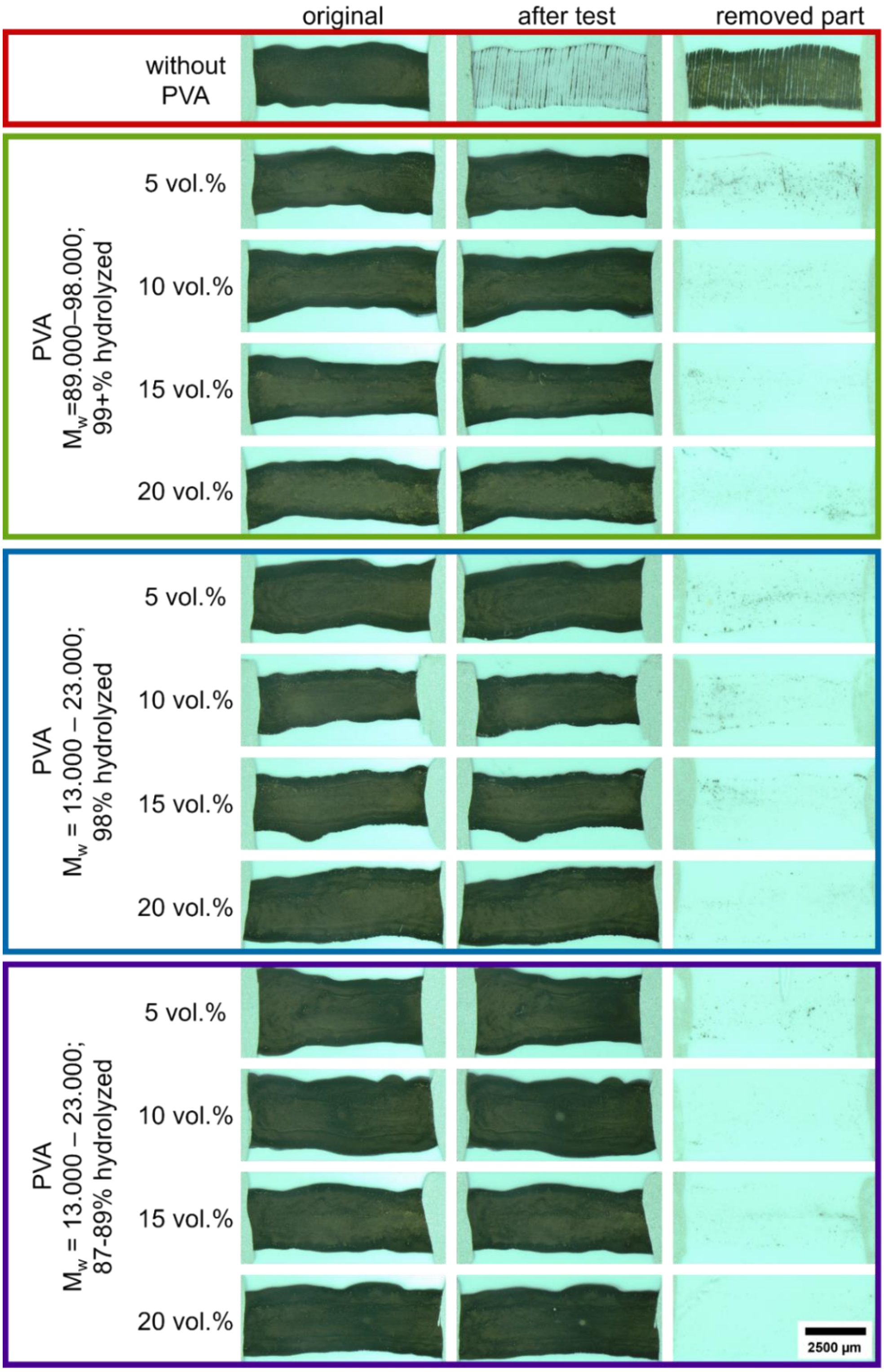

Figure S2: Adhesion tests on drop-casted samples with different PVA types and contents. Shown are optical micrographs of the sample before and after the test, and the removed part of the material on the tape.

3. Effect of drying time, type, and PVA content on the resistivity of drop-casted samples.

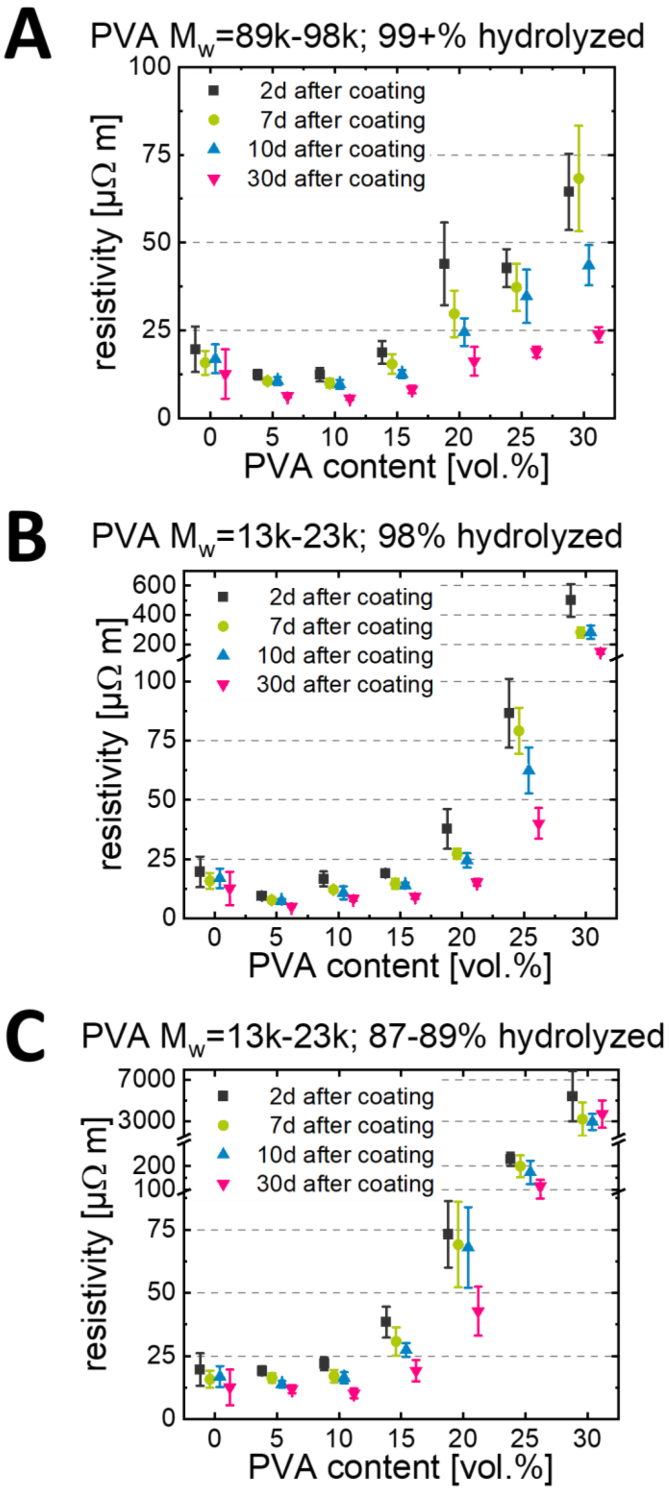

Figure S3: Drop casted samples of hybrid inks with different PVAs and different PVA contents of 0, 5, 10, 15, 20, 25, and 30 vol.%. **A** PVA M_w = 89k-98k; 99+% hydrolyzed; **B** with PVA M_w = 13k-23k; 98%; **C** PVA M_w = 13k-23k; 87-89% hydrolyzed.
